# Right-sided infective endocarditis with coronary sinus vegetation

**DOI:** 10.1186/s12872-018-0845-x

**Published:** 2018-06-04

**Authors:** Guang Song, Jing Zhang, Xintong Zhang, Huan Yang, Wanying Huang, Ming Du, Ke Zhou, Weidong Ren

**Affiliations:** 1 Department of Ultrasound, Shengjing Hospital of China Medical University, Shenyang, China; 2 Department of Pathology, Shengjing Hospital of China Medical University, Shenyang, China; 3 Department of Radiology, Shengjing Hospital of China Medical University, Shenyang, China; 4 Department of Cardiac Surgery, Shengjing Hospital of China Medical University, Shenyang, China

**Keywords:** Coronary sinus, Infective endocarditis, Vegetation, Surgery, Echocardiography

## Abstract

**Background:**

Infective endocarditis (IE) is a rare disease with high mortality. Right-sided IE accounts for 5–10% of cases of IE. The tricuspid valve is most commonly affected, oppositely in coronary sinus (CS). The diagnoses, treatments and outcomes of CS vegetation has not been summarized yet.

**Case presentation:**

We present a 71-year-old man complained of cough and fever. Transthoracic echocardiography revealed the aneurysmal dilated CS with the band medium-echo mobile structure. A sinus venosus atrial septal defect has been detected. He had a persistent left superior vena cava which drained the right atrium via the aneurysmal dilated CS. Blood cultures were positive for *Staphylococcus aureus*. After intravenous antibiotic therapy, he had the symptom of dyspnea. The suspicious diagnosis is recurrent septic lung emboli which was confirmed by thoracic contrast enhanced computed tomography. The thoracotomy was performed to repair the atrial septum and remove the CS vegetation. Ten days later, the patient was discharged with only mild cough.

**Conclusion:**

Both positive blood cultures and echocardiography are major criteria in right-sided IE with CS vegetation. Current treatment options of CS vegetation include medical therapy and surgery. The surgical strategy for CS vegetation should be individualized, due to the controversial indications and optimum time of surgery. Most people have a good prognosis after proper treatment.

**Electronic supplementary material:**

The online version of this article (10.1186/s12872-018-0845-x) contains supplementary material, which is available to authorized users.

## Background

Infective endocarditis (IE) is a rare disease with prevalence ranging from 3 to 10 per 100,000 person-years [1]. The average age of patients with infective endocarditis are increasing from 40 shifting to 70 [2]. Due to its high mortality (20–25%) in the past two decades, IE is now the third or fourth most common life-threatening infection syndrome [1, 3, 4]. According to its principal associated complications in different clinical scenarios, IE could be divided into several types, including the left-sided IE, right-sided IE, prosthetic valve IE, electronic devices IE, and so on. Right-sided IE accounts for 5–10% of cases of IE [5]. The tricuspid valve is most commonly affected, oppositely in coronary sinus (CS) [5]. Herein, we present the case of a 71-year-old man with right-sided IE and CS vegetation.

## Case presentation

**Fig. 1:**
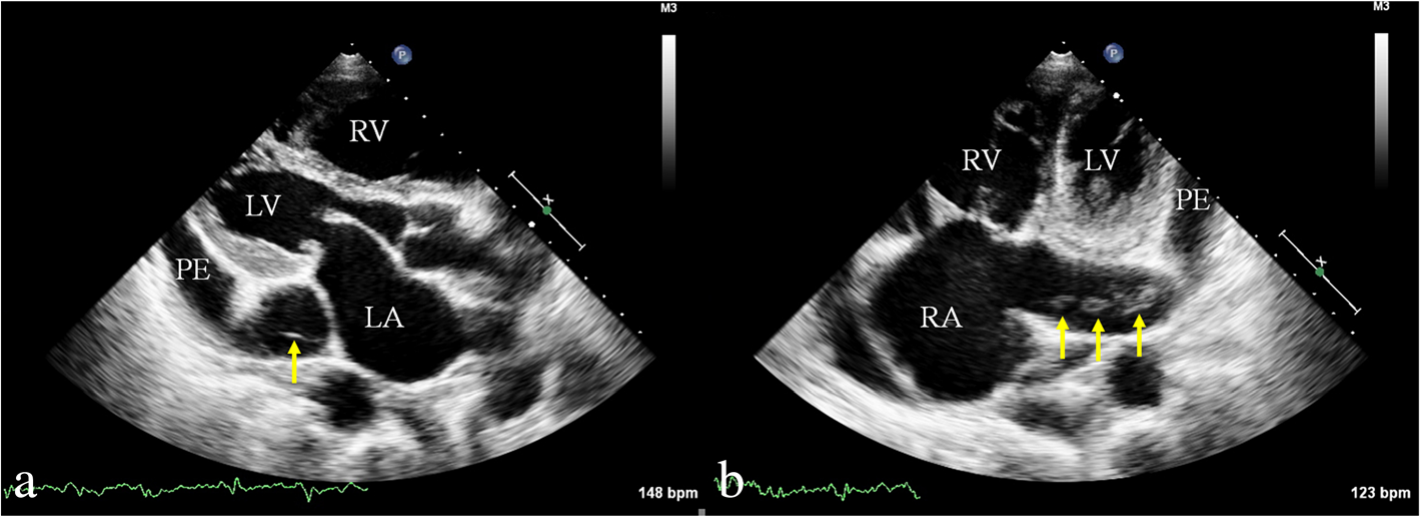
The aneurysmal dilated coronary sinus with the banded medium-echo mobile structure (yellow arrow). **a** in the parasternal left ventricle long-axis view. **b** In the modified apical 4-chamber view. CS: Coronary sinus; LA: Left atrium; LV: Left ventricle; PE: Pericardial effusion; RV: Right ventricle

**Fig. 2:**
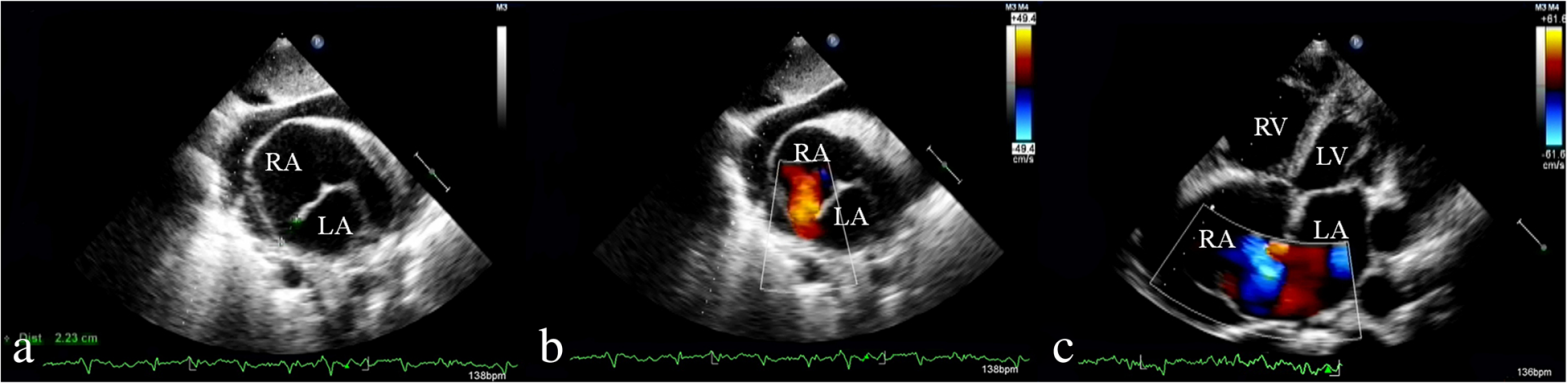
Sinus venosus atrial septal defect. **a** The size of echo drop is 22.3 mm. **b, c** bi-directional shunt has been detected between right atrium and left atrium. LA: Left atrium; LV: Left ventricle; RA: Right atrium; RV: Right ventricle

**Fig. 3:**
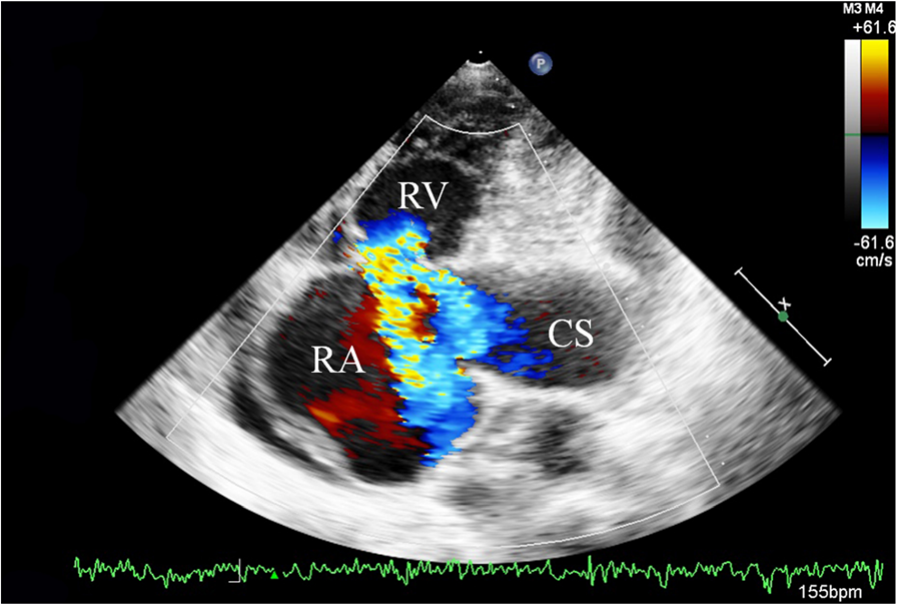
Severe tricuspid regurgitation has been revealed, part of which drained into the coronary sinus. CS: Coronary sinus; RA: Right atrium; RV: Right ventricle

**Fig. 4:**
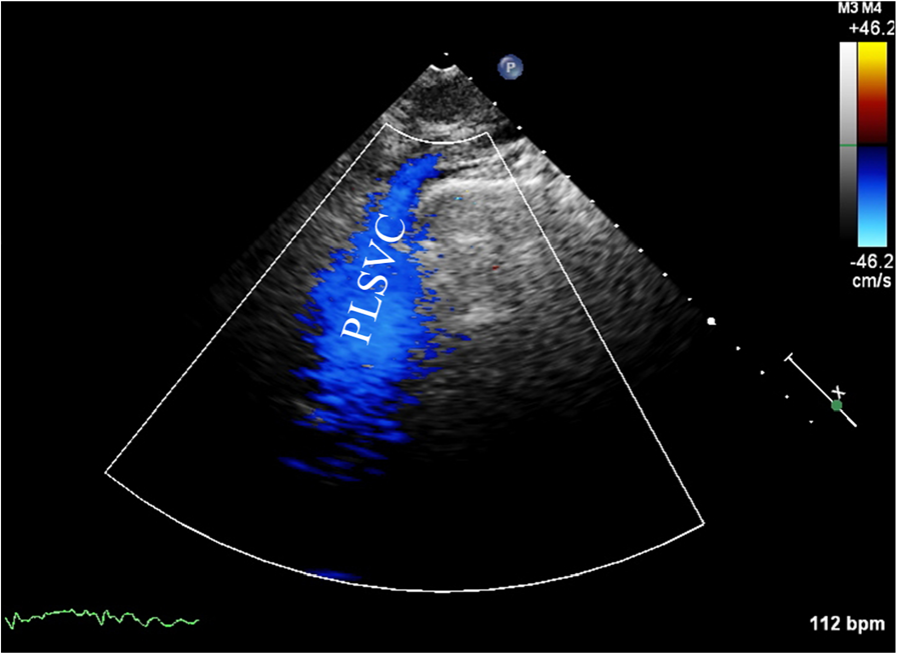
The persistent left superior vena cava has been revealed in the suprasternal long axis view of aortic arch. PLSVC: Persistent left superior vena cava

**Fig. 5:**
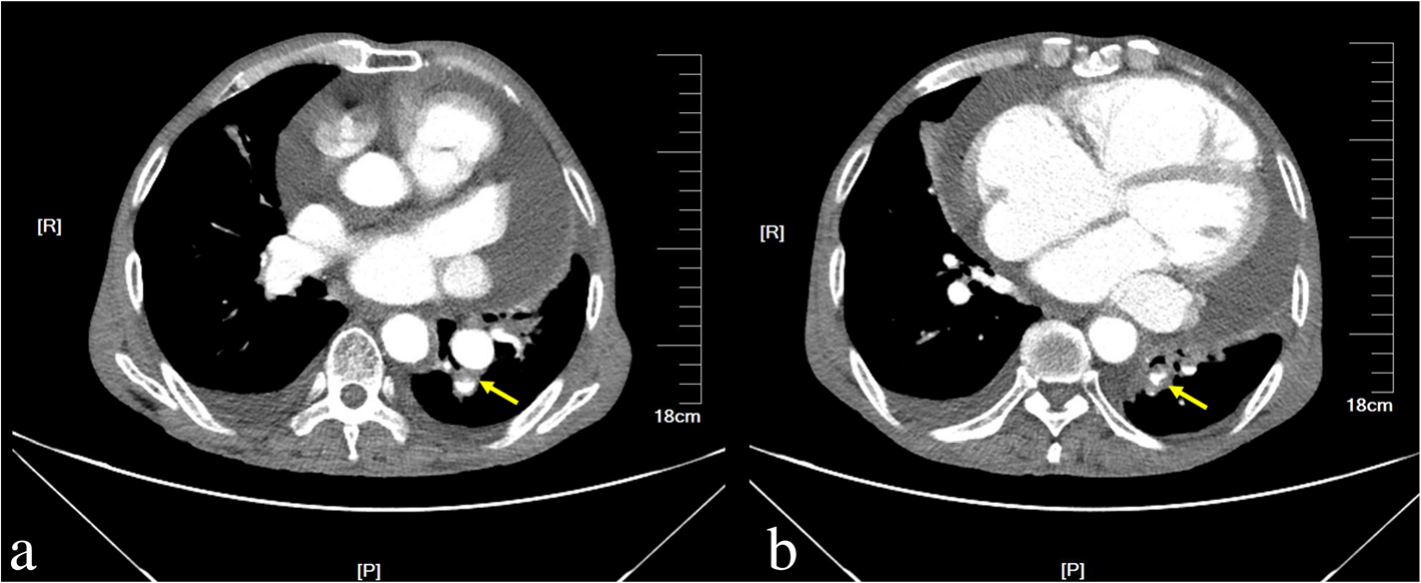
Emergency thoracic contrast enhanced computed tomography revealed pulmonary embolism. **a**, **b** filling defects in the branches of the left lower pulmonary artery (yellow arrow)

**Fig. 6:**
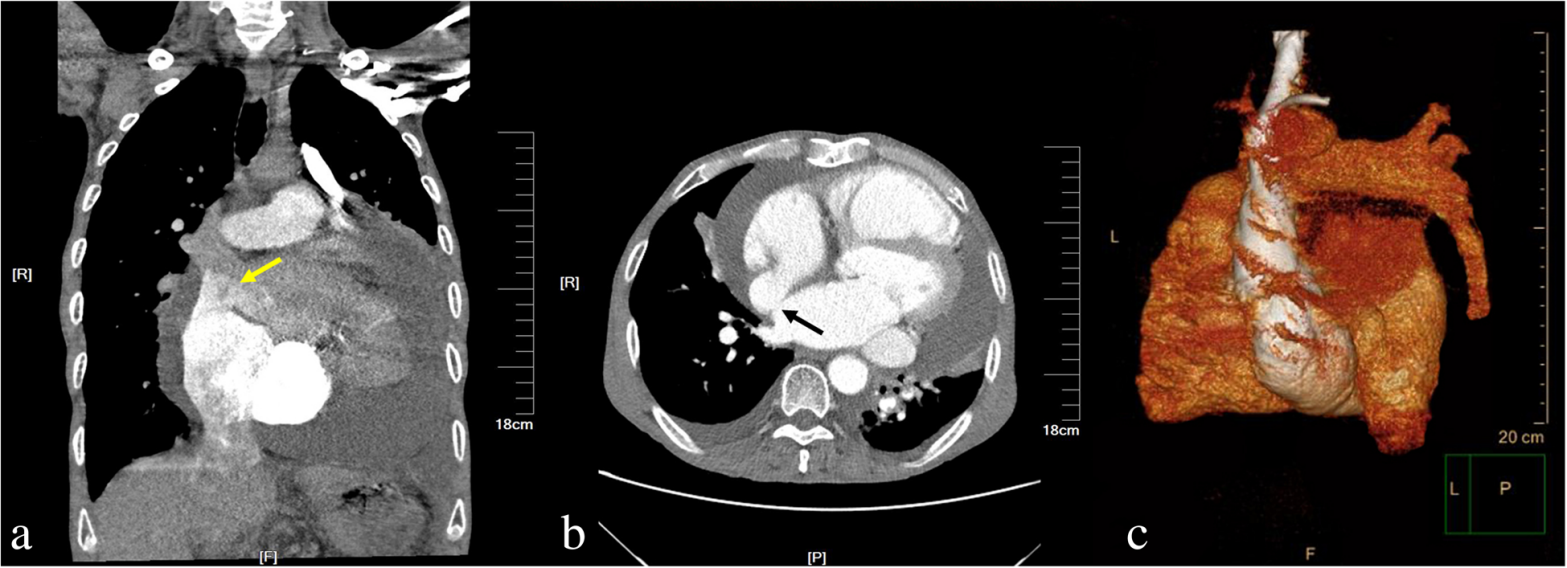
Emergency thoracic contrast enhanced computed tomography revealed cardiovascular anomalies. **a** Contrast agent slightly appeared in left atrium (yellow arrow) when the right heart was enhancing. **b** Interruption was visible between right atrium and left atrium. **c** A persistent left superior vena cava drained into the right atrium through the aneurysmal dilated coronary sinus

**Fig. 7:**
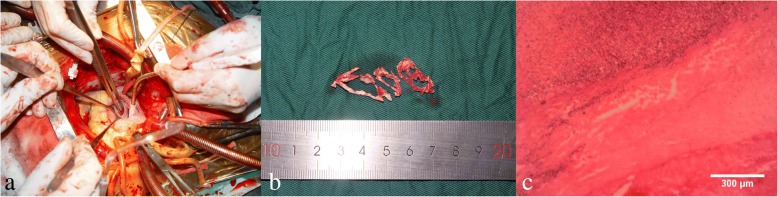
**a** Thoracotomy was performed to repair the atrial septum and remove the coronary sinus vegetation. **b** Photograph of the gross specimen showed a netlike vegetation which was removed from the coronary sinus. The vegetation was mixed with white and dark red. **c** Histologic sectioning revealed that vegetation contained a large number of necrotic material and neutrophils


A 71-year-old previously healthy man complained of cough and fever for 1 month. At first, he was evaluated at his local hospital, where he was believed to have pneumonia. During hospitalization, he got sudden chest pain and hemoptysis which were similar as the symptoms of the pulmonary embolism. However, lower extremity doppler ultrasound didn’t found any sign of thrombus. He presented to our hospital for a definite diagnosis. On day 1, his pulse rate was 106 beats/minute, blood pressure was 134/83 mmHg, and his temperature was 38.3 °C on examination. There were no murmurs on auscultation of the heart. Laboratory testing revealed a white blood cell count of 21,000/μL (neutrophils 74.5%), hemoglobin of 11.5 g/dL, and platelets of 192,000/μL. Also, the C-reactive protein level is 74 mg/L, and erythrocyte sedimentation rate is 66 mm/h. The coagulation function is normal. The electrocardiogram showed sinus tachycardia without other abnormalities. On day 2, transthoracic echocardiography (TTE) revealed the aneurysmal dilated CS (diameter: 38 mm) with the band medium-echo mobile structure in the parasternal left ventricle long-axis view (Fig. 1a; Additional file 1: Movie 1). In the modified apical 4-chamber view, the band medium-echo mobile structure (40 × 12 mm) could be observed in the aneurysmal dilated CS (Fig. 1b; Additional file 2: Movie 2). A sinus venosus atrial septal defect (ASD) with bi-directional shunt has been detected near the entrance of superior vena cava in the right atrium (Fig. 2a, b, c). Part of severe tricuspid regurgitation drained into the CS (Fig. 3). The persistent left superior vena cava has been revealed in the suprasternal long axis view of aortic arch (Fig. 4). Enlarged right heart, pericardial effusion, dilated inferior vena cava may indicate dysfunctional right heart. Moderate pulmonary artery hypertension also has been revealed. On day 4, blood cultures were positive for *Staphylococcus aureus* which is methicillin sensitive. We highly suspect that this is IE with CS vegetation. So, he got intravenous antibiotic therapy which lasted 2 weeks during hospitalization. Cloxacillin is given by intravenous injection as 12 g/day in 4–6 doses. However, on day 6, the patient had symptoms of dyspnea and chest pain. We repeated the blood cultures which were also positive. We believed these symptoms caused by recurrent septic lung emboli. Emergency thoracic contrast enhanced computed tomography was performed and revealed filling defects in the branches of the left lower pulmonary artery (Fig. 5a, b). Sinus venosus ASD has been confirmed (Fig. 6a, b). A persistent left superior vena cava drained into the right atrium through the aneurysmal dilated CS (Fig. 6c). On day 8, thoracotomy was performed (Fig. 7a). A photograph of the gross specimen showed a netlike vegetation which was removed from the CS (Fig. 7b). The vegetation was mixed with white and dark red. Histologic sectioning revealed that vegetation contained a large number of necrotic material (Fig. 7c). After surgery, his condition became stable. On day 18, the blood culture and other laboratory testing normalized. The patient was discharged with only mild cough. After he got home, he also had two-week antibiotherapy in his local hospital. Two months later, he came to the outpatient department for follow-up. He was doing well without any complications. TTE only revealed the dilated CS and pericardial effusion.

**Additional file 1**: **Movie 1** Transthoracic echocardiography revealed the aneurysmal dilated coronary sinus with the band medium-echo mobile structure in the parasternal left ventricle long-axis view. (AVI 1089 kb)



**Additional file 2**: **Movie 2** Transthoracic echocardiography revealed the band medium-echo mobile structure could be observed in the dilated coronary sinus in the modified apical 4-chamber view. (AVI 564 kb)


## Discussions and Conclusions

Unlike the left-sided IE mainly occurred on the aorta or mitral valve, right-sided IE could involve the tricuspid valve, pulmonary valve, eustachian valve, interventricular septum, right ventricular free wall, or CS. The right-sided IE with CS vegetation, just like our case, is extremely rare. So far, we have found 7 cases describing CS vegetation (Table 1) [6–11]. There were 3 men and 4 women. Ages ranged from 23 years to 71 years; mean age 39.6 ± 19.8 years. Right-sided IE usually occurs in intravenous drug abusers or patients with human immunodeficiency virus [10, 11], cardiac device infection, central venous catheter, congenital heart disease, and hemodialysis [8]. These risk factors may cause cardiac endothelial damage [9]. The most common symptom of these patients is fever. Due to the possibility of abscission of vegetation, right-sided IE with CS vegetation could present with a complication, particularly septic shock or pulmonary embolism (hemoptysis).

**Table 1:** Summary of literature involving right-sided IE with CS vegetation

No.	First Author	Year	Sex, age	Symptoms	First modality for diagnosis	Length × width of vegetation (mm)	Associated cardiovascular anomalies	Blood culture	Treatments	Outcomes
1	Kasravi [6]	2004	M, 31y	Fever, chills, nausea, vomiting, myalgias, neck stiffness	TTE	> 27^a^× 6^a^	CACSF, vegetation extends to RA	Positive for *Staphylococcus aureus*	Antibiotic	Alive
2	Gill [7]	2005	M, 37y	Fever, weight loss	TEE	14 × 7	CACSF	Positive for Streptococcus	Antibiotic	Alive
3	Kwan [8]	2014	F, 23y	Fever	TTE	14 × 2^a^	Vegetation extends to RA	Positive for Acinetobacter baumanii	Antibiotic	Alive
4	Takashima [9]	2016	F, 64y	Fever, fatigue, loss of appetite, septic shock	TTE	17 × 9^a^	CACSF, vegetations on the mitral and aortic valves with moderate regurgitation, heart failure	Negative	Surgery	Died
5	Kumar [10]	2017	F, 23y	Septic shock	TEE	30 × 5	Vegetation on the Eustachian valve in the RA	/	Antibiotic	Alive
6	Theodoropoulos [11]	2017	F, 28y	Fever, sweat malaise, hemoptysis, dyspnea	TTE	15^a^× 8^a^	Tricuspid valves with moderate regurgitation	Positive for Streptococcus	Antibiotic	Alive
7	Our case	2017	M, 71y	Fever, cough, chest pain, hemoptysis, dyspnea	TTE	40 × 12	ASD, PLSVC, tricuspid valves with severe regurgitation	Positive for Staphylococcus aureus	Antibiotic, surgery	Alive

*ASD* Atrial septal defect, *CACSF* Coronary artery-coronary sinus fistula, *PLSVC* Persistent left superior vena cava, *RA* Right atrium, *TEE* Transesophageal echocardiography, *TTE* Transthoracic echocardiography

^a^: measured from the figures in the literature

The first modality for diagnosis is TTE (71%) and transesophageal echocardiography (29%). Echocardiography is crucial to diagnosis. TTE is a first line imaging study in the diagnosis of IE at present [5]. In suspected IE, TTE has a moderate sensitivity (75%) and high specificity (> 90%) in detecting IE with vegetation [12]. In patients with an equivocal or negative TTE, but high clinical likelihood of infective endocarditis, transesophageal echocardiography is necessary due to the higher sensitivity (> 90%). Each of three positive echocardiographic findings which include vegetation, cardiac abscess, and new valvular regurgitation could provide sufficient evidence of IE [13]. In particular, vegetation is the landmark lesion of IE. Vegetation in the CS has some characters: First, vegetation is usually isolated, may not affect other valves. Second, the CS always dilated. Third, vegetation in the CS is usually big (length > 10 mm), shaped like a tubule mass. Our case is the only netlike one. Most of CS vegetations are mobile. Echocardiography could detect of the associated cardiovascular anomalies, including coronary artery-CS fistula [6, 7, 9], ASD, and valvular regurgitation. Echocardiography also could provide information about severity of the valve lesion, and assess the left/right ventricular function [14].

Blood culture is crucial as a major criterion for the diagnosis of right-sided IE. In these seven patients, about 71% (5/7) of blood cultures were positive. Pathogenic bacteria included *Staphylococcus aureus* (2), *Streptococcus* (2), and *Acinetobacter baumanii* (1). Similar results have been found by previous studies which revealed that *Staphylococcus aureus* was the most common cause of right-sided IE [15, 16]. *Staphylococcus aureus* infection has been shown to be an independent predictor of mortality from IE [17]. Doctor must pay special attention to this kind of microorganism because *Staphylococcus aureus* has a higher mortality (51%) than others (31%) [18]. The high mortality may due to its complicated large vegetations, invasive valve damage, and embolic symptom [19]. The negative result of blood culture has a incidence of 2.5–31%, which could delay diagnosis and the initiation of treatment [20].

Current treatment options of IE include medical therapy and surgery. Most of the patients with CS vegetation (86%) received antibiotic therapy. Medical therapy is the primary treatment strategy [5]. On an empirical basis, antibiotics should be started as soon as blood cultures have been acquired, but doctors could also await result of blood culture if the condition of patient is stable [14]. Treatment for at least 4–6 weeks is usually necessary. Although undertaken in 40–50% of patients with IE [3, 21], the necessity and indications of surgery main controversial. According to previous studies and guidelines, indications for surgery of IE have been summarized [22–24] (Table 2). The purpose of surgery is to eradicate the infection and achieve hemodynamic correction. The reason for surgery in our case is as follow: (1) Patients may have recurrent septic pulmonary emboli on his sixth day in hospital. (2) Patients with severe tricuspid regurgitation and dysfunctional right heart. (3) The size of vegetation larger than 10 mm. (4) The patient has a risk of paradoxical embolism due to associating ASD with bi-directional shunt. Cerebrovascular complications, causing by paradoxical embolism, could decease the quality of life in the long term. The optimum time of surgery remains indistinct. Previous study revealed that early surgery should be considered early if *Staphylococcus aureus* is suspected [23]. Half of surgeries were performed in the acute phase, the other half in the convalescent phase [25].

**Table 2:** Indications for surgery of IE according to the previous studies and guidelines [22–24]

1. Patients with persistent infection who do not respond to antibiotic therapy beyond 2 weeks, except for specific pathogens that aggressive treatment should be considered early in the course of the disease (e.g. Staphylococcus aureus, Gram negative fungi); Perivalvular extension: abscesses, fistulas.	
2. Patients with recurrent septic pulmonary emboli, confirmed by computed tomography pulmonary angiogram.	
3. Patients with massive or worsening tricuspid regurgitation (> 2+/4+) contributing to deteriorating right (and subsequently impending left) ventricular heart failure; evaluated by echocardiography.	
4. Patients in septic shock and documented right-sided IE (indication for emergency operation).	
5. When the size of a vegetation increases or persists in spite of antibiotic management at > 10 mm.	
6. New-onset acute or worsening renal and/or hepatic failure.	
7. Patients with right-sided IE who develop a secondary (right- or left-sided) valve endocarditis (multivalvular involvement).	
8. Following failure or complications of percutaneous removal of infected intracardiac wires.	
9. Complicated prosthetic valve IE: Caused by Staphylococcus aureus.	

*IE* Infective endocarditis

Prognosis of right-sided IE is usually well. Previous study revealed the mortality of right-sided IE is 12% in-hospital patients [26], and 0–7.3% for surgical patients [27, 28]. In these seven patients with CS vegetation, the in-hospital mortality is 14%. Most of the patients have an uneventful recovery. Only one patient died due to multiple organ failure after her surgery.

Both positive blood cultures and echocardiography are major criteria in right-sided IE with CS vegetation. Current treatment options of right-sided IE include medical therapy and surgery. The surgical strategy for right-sided IE patients with CS vegetation should be individualized, due to the controversial indications and optimum time of surgery. Most people have a good prognosis after proper treatment.

## Abbreviations

ASD: Atrial septal defect
CS: Coronary sinus
IE: Infective endocarditis
TTE: Transthoracic echocardiography
